# Transposon-Derived Non-coding RNAs and Their Function in Plants

**DOI:** 10.3389/fpls.2018.00600

**Published:** 2018-05-03

**Authors:** Jungnam Cho

**Affiliations:** The Sainsbury Laboratory, University of Cambridge, Cambridge, United Kingdom

**Keywords:** transposable elements, domestication, small RNA, long non-coding RNA, microRNA target mimic

## Abstract

Transposable elements (TEs) are often regarded as harmful genomic factors and indeed they are strongly suppressed by the epigenetic silencing mechanisms. On the other hand, the mobilization of TEs brings about variability of genome and transcriptome which are essential in the survival and evolution of the host species. The vast majority of such controlling TEs influence the neighboring genes in *cis* by either promoting or repressing the transcriptional activities. Although TEs are highly repetitive in the genomes and transcribed in specific stress conditions or developmental stages, the *trans*-acting regulatory roles of TE-derived RNAs have been rarely studied. It was only recently that TEs were investigated for their regulatory roles as a form of RNA. Particularly in plants, TEs are ample source of small RNAs such as small interfering (si) RNAs and micro (mi) RNAs. Those TE-derived small RNAs have potentials to affect non-TE transcripts by sequence complementarity, thereby generating novel gene regulatory networks including stress resistance and hybridization barrier. Apart from the small RNAs, a number of long non-coding RNAs (lncRNAs) are originated from TEs in plants. For example, a retrotransposon-derived lncRNA expressed in rice root acts as a decoy RNA or miRNA target mimic which negatively controls miRNA171. The post-transcriptional suppression of miRNA171 in roots ensures the stabilization of the target transcripts encoding SCARECROW-LIKE transcription factors, the key regulators of root development. In this review article, the recent discoveries of the regulatory roles of TE-derived RNAs in plants will be highlighted.

## Introduction

Transposable elements (TEs) are the major constituent of many eukaryotic genomes. Especially in the cereal crops (e.g., barley, wheat, and maize), more than 80% of their genomes are made up of transposons (Tenaillon et al., 2010). TEs are classified to two major classes depending on their modes of transposition; class I and class II (Feschotte et al., 2002; Wicker et al., 2007). Class I TEs, also known as retrotransposons, move through RNA intermediates that are later converted to cDNAs, creating extra copies in the genome. The long terminal repeat (LTR) retrotransposons and the long interspersed nuclear elements (LINEs) are the two main types of retrotransposon. Both LTR retrotransposons and LINEs are autonomous elements since they encode for the proteins necessary for transposition, while those that depend on the autonomous elements such as large retrotransposon derivatives (LARDs), terminal repeat retrotransposons in miniature (TRIMs) and short interspersed nuclear elements (SINEs) are non-autonomous retrotransposons. Unlike class I, class II transposons, or DNA TEs, are excised from one location and insert to another genomic position by the transposase protein which is encoded within DNA TEs. Class II TEs include another subclass, Helitrons, which replicate through rolling circle amplification. In many plant genomes, retrotransposons are more abundant compared to class II elements. Particularly, the LTR retrotransposons are the predominant families of TEs in many plants (Vitte et al., 2017). The replication cycle of the LTR retrotransposons initiates with transcription of genomic copy by the host’s RNA polymerase (Pol) II. The mRNAs of LTR retrotransposons are subjected to both translation and reverse-transcription (Grandbastien, 1998). Autonomous LTR retrotransposons produce multiple proteins including GAG, aspartic protease, reverse-transcriptase, RNase H and integrase which are required for the completion of retrotransposition cycle. As a result of reverse-transcription, the linear and double-stranded DNA is produced which is known as extrachromosomal DNA (ecDNA). The ecDNAs are then transported back to the nucleus and integrate to genomic chromosomal DNA by the integrase protein.

Since TE mobilization can be mutagenic, the host genomes have evolved elaborate mechanisms to suppress their activities (Matzke and Mosher, 2014). TEs are primarily repressed by the epigenetic silencing pathways including histone modification and DNA methylation. In plants, the RNA-directed DNA methylation (RdDM) pathway plays a central role in TE silencing. Genomic regions marked by DNA methylation are recognized by the plant-specific RNA polymerase, RNA PolIV, which transcribes relatively short stretches of RNAs (Blevins et al., 2015; Li et al., 2015; Zhai et al., 2015). The transcribed RNAs are then duplexed by the RNA-dependent RNA polymerase (RDR) 2 and subsequently sliced to 24 nucleotide (nt) small interfering (si) RNAs by the DICER-like (DCL) 3. These 24 nt-siRNAs are bound by the ARGONAUTE (AGO) 4 proteins and interact with the nascent RNA transcribed by the RNA PolV. AGO4 then recruits multiple proteins including SU(VAR)3-9 HOMOLOG (SUVH) 4/5/6 and DOMAINS REARRANGED METHYLASE (DRM) 1/2 that mediate repressive histone modification (H3K9me2) and DNA methylation, respectively, thus contributing to reinforcement of the silenced state of TE chromatins (Zilberman et al., 2004; Tran et al., 2005; Zhong et al., 2014). TEs escaped from silencing or newly introduced to the genome are recognized by the RDR6-RdDM pathway that post-transcriptionally degrades TE mRNAs. RNA PolII-transcribed TE mRNAs are processed to 21 or 22 nt-siRNAs by the RDR6 and DCL2/4 (Creasey et al., 2014). These 21 or 22 nt-siRNAs associate with AGO1 and target TE mRNAs for degradation. Intriguingly, TE-associated siRNAs can also interact with non-TE target transcripts exerting certain regulatory roles in various biological processes. In mammals, PIWI-interacting RNAs regulate a large number of mRNAs and long non-coding RNAs (lncRNAs) in testis, suggesting widespread regulatory roles of TE-derived small RNAs in both plants and animals (Watanabe et al., 2015).

In addition to siRNAs, many plant miRNAs have been suggested to be evolved from TEs (Piriyapongsa and Jordan, 2008; Li et al., 2011). Although miRNAs are distinct from siRNAs in origin and biogenesis by definition (Borges and Martienssen, 2015), the categorization of small RNAs identified by deep sequencing has not been done with sufficient precision. In fact, considerable number of siRNAs are mis-annotated to miRNAs (McCue et al., 2012). Nonetheless, multiple lines of evidence indicate that TEs have co-opted to miRNAs since the repeated sequences associated with TEs can readily form RNA hairpin structures that can be subsequently processed by miRNA biogenesis pathways (Piriyapongsa and Jordan, 2008; Li et al., 2011). Moreover, the vast majority of lncRNAs are originated from TEs in mammalian as well as plant genomes (Kelley and Rinn, 2012; Liu et al., 2012; Kapusta et al., 2013), suggesting dynamic evolutionary exaptation of TEs in the form of RNA. In the following two sections, several examples of TE domestication to functionally relevant regulatory RNAs in plants will be explained.

## Transposon-Derived Small RNAs

### TE-siRNA in Stress Response

**FIGURE 1:**
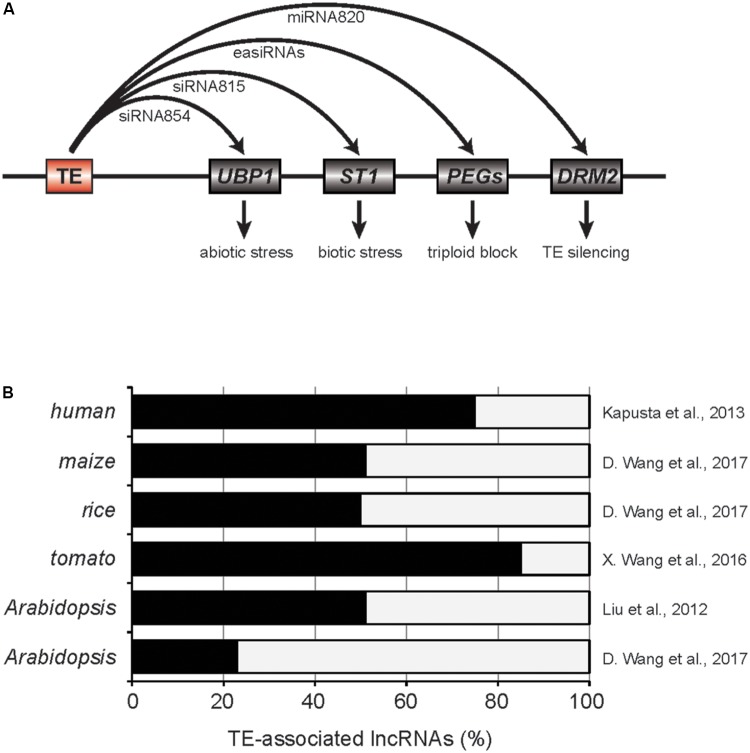
TE-associated small RNAs and long non-coding RNAs. **(A)** Roles of TE-derived small RNAs in plants. Red and gray boxes are TE and genes, respectively. PEGs, paternally expressed imprinted genes. **(B)** The fractions of lncRNAs associated to TEs. Related references are provided on the right.

In the mutant of *Decreased DNA methylation 1* (*DDM1*), a gene encoding ATP-dependent chromatin remodeler in *Arabidopsis*, global DNA methylation level is dramatically reduced, thereby numerous TEs are reactivated (Vongs et al., 1993; Creasey et al., 2014). A large fraction of those reactivated TEs are accompanied with the production of 21 or 22 nt-siRNAs, known as epigenetically activated siRNAs (easiRNAs) (Creasey et al., 2014). The easiRNAs target TE transcripts for cleavage ensuring the silencing of TEs at the post-transcriptional step. Interestingly, a subset of the easiRNAs in *ddm1* mutants can interact with genic mRNAs reducing their expression levels. For example, siRNA854 is one of the easiRNAs generated in *ddm1* mutant and produced from *Athila6A* TE. It targets 3′ UTR of *UBP1* transcript which encodes a stress granule protein (**Figure 1A**; McCue et al., 2012). Using the multiple reporter gene constructs containing the 3′ UTR of *UBP1*, it was demonstrated that siRNA854 represses non-TE targets as well. The expression levels of *Athila6A* and siRNA854 are increasingly upregulated in multiple generations of *ddm1* mutation. For example, the plants with *ddm1* homozygous mutation for six generations (*ddm1* F6) have higher levels of siRNA854 compared to *ddm1* F2. The *ubp1* mutants show strong susceptibility to osmotic stress and similar phenotype was also observed in *ddm1* F6 but not in *ddm1* F2 plants. Therefore, the targeting of *Athila6A*-derived siRNA854 to *UBP1* transcript and alteration of resistance to abiotic stresses provides insight into how TEs adapted to changing environment in plants. In addition to *UBP1*, *Athila6A*-derived easiRNAs can target other genic mRNAs including *AMS* and *HHP2* (McCue et al., 2013). Several of those targets were experimentally validated for the easiRNA-mediated repression by the short tandem target mimic (STTM) transgenic approach, however, the biological relevance of this regulation is yet to be answered.

A more recent paper by Zhang et al. (2016) suggested that TE-siRNA815 in rice can induce *de novo* DNA methylation in the target gene loci through RdDM pathway (**Figure 1A**). Two allelic transcription factor genes, *WRKY45-1* and *WRKY45-2*, were previously shown to have opposing effects in the resistance to *Xanthomonas oryzae* pv. *oryzae* (*Xoo*). *WRKY45-1* allele produces TE-siRNA815 from WANDERER_OS-type DNA TE located in the intron. TE-siRNA815 then recognizes the complementary sequence and deposits DNA methylation through RdDM pathway in the intron of *ST1* locus, which is critical in the resistance against *Xoo*. On the other hand, *WRKY45-2* allele lacks such siRNA producing region that ensures stable *ST1* expression contributing to the pathogen resistance.

### TE-siRNA in Hybridization Barrier

Since easiRNAs are mainly produced in the epigenetic mutants, it has been questioned if easiRNAs have a function in natural conditions. Two recent studies answered this question by demonstrating the roles of easiRNAs in the hybridization barrier in *Arabidopsis* (Borges et al., 2018; Martinez et al., 2018). The vegetative nuclei of pollen grains have reduced activity of *DDM1* and numerous TEs are reactivated (Slotkin et al., 2009). Pollen-specific miRNA845b recognizes the conserved sequence [primer-binding site (PBS)] in the LTR retrotransposons activated in pollen and triggers the initial cleavage of LTR-TE mRNAs followed by the production of easiRNAs (Borges et al., 2018). Higher dosage of the paternal genome brings higher amount of easiRNAs in fertilization which gives rise to the unbalanced gametic siRNAs and ultimately seed failure (triploid block, Martinez et al., 2018). When the paternal easiRNA levels were suppressed by *poliv* mutation, the triploid blockage phenotype was partly restored, indicating a critical role of the paternal easiRNAs in the hybridization barrier (Martinez et al., 2018). The exact mechanism for the easiRNA-mediated triploid block is still unclear, however, it was suggested that the excess paternal easiRNAs might interfere with DNA methylation establishment around the paternally expressed imprinted genes by hijacking PolV-transcribed nascent transcripts (**Figure 1A**).

### TE-Small RNA as Anti-silencing Factor

Transposable elements have often domesticated to miRNA genes in plants (Li et al., 2011). One example is miRNA820 of rice. miRNA820 is 22 or 24 nt in size and is originated from the internal region of CACTA DNA TE (Nosaka et al., 2012). Interestingly, miRNA820 targets the transcripts of *DRM2* that encodes a *de novo* DNA methyltransferase (**Figure 1A**). The targeting of miRNA820 to *DRM2* is evolutionarily conserved in the *Oryza* genus and the repression of *DRM2* gene results in strong reduction in DNA methylation and transcriptional upregulation of many TEs. Therefore, miRNA820 can be seen as an anti-silencing factor encoded within a TE that works at the post-transcriptional level. Similarly, UBP1 is the *Arabidopsis* homolog of TIA-1 in mammals which is known to suppress the viral translation of Tick-Borne Encephalitis Virus (Albornoz et al., 2014). McCue et al. (2013) have also shown that UBP1 protein forms the cytoplasmic stress granules in abiotic stress condition or when heterochromatic TE silencing is released, for instance in *ddm1* mutant. As is the case in mammals that TIA-1 inhibits the viral translation, the level of GAG protein encoded in *Athila6A* was elevated in *ddm1 rdr6 ubp1* triple mutants (McCue et al., 2013). This data supports the notion that UBP1 acts on the activated TE mRNAs to suppress their translation and therefore TE-siRNAs are the repressors of the host TE silencing mechanism.

## Transposon-Derived lncRNAs

There is emerging evidence of TE domestication to lncRNAs in both mammalian and plant genomes (**Figure 1B**; Kelley and Rinn, 2012; Kapusta et al., 2013; Johnson and Guigo, 2014; Liu et al., 2015; Wang et al., 2015, 2016; Quattro et al., 2017). LncRNA can be defined as a transcript of at least 200 bp in size but has low protein-coding potential (Liu et al., 2015). It is well-studied that lncRNAs perform various cellular function including the recruitment of the epigenetic regulators to target chromatin or the sequestration of miRNAs (Franco-Zorrilla et al., 2007; Heo and Sung, 2011; Csorba et al., 2014). In the past decade, transcriptomic analyses have dramatically expanded the catalog of the lncRNAs in various tissues and stress conditions of many plant species. Despite the large number of plant lncRNAs identified so far, however, the biological roles are still largely unexplored. In this section, the current status of plant TE-lncRNA studies will be discussed.

### TE-lncRNA in Stress Response

Since many TEs in plants possess stress-responsive *cis*-acting elements within them (Paszkowski, 2015), TE-lncRNAs often appear in specific stress conditions (Liu et al., 2012; Quattro et al., 2017; Wang et al., 2017). In a recent report, Wang et al. (2017) interrogated the lncRNAs in *Arabidopsis*, rice and maize under various abiotic stresses. There was a large discrepancy in TE families that TE-lncRNAs are originated from; RC/Helitron in *Arabidopsis*, MITEs in rice and LTR retrotransposons in maize were predominantly overrepresented in TE-lncRNAs (Wang et al., 2017). Particularly, TE-lncRNA11195 in *Arabidopsis* contains an LTR-type retrotransposon and is activated after abiotic stresses or ABA treatment. The deletion of the LTR sequence compromised the ABA responsiveness, suggesting that LTR sequence conferred the stress responsiveness to TE-lncRNA11195 (Wang et al., 2017). TE-lncRNA11195 was tested for its role in stress response using T-DNA insertional mutants. Interestingly, two independent mutant lines showed marked increase in resistance to abscisic acid (ABA) in root elongation and shoot fresh weight (Wang et al., 2017). TE-lncRNAs in tomato were also described to be responsive for both abiotic and biotic stresses (Wang et al., 2015), however, the biological function has not been investigated as yet.

### TE-lncRNA and Development

In mammals, the majority of lncRNAs are derived from TEs and exhibit strong tissue-specific expression pattern (Kelley and Rinn, 2012; Kapusta et al., 2013). Similarly, TE activation in plants is associated with specific development stages potentiating the emergence of tissue-specific TE-lncRNA (Hsieh et al., 2009; Slotkin et al., 2009; Baubec et al., 2014; Cho and Paszkowski, 2017). Recently, Cho and Paszkowski (2017) have investigated the expression pattern of TEs in various rice tissues and identified a retrotransposon-derived transcript called *MIKKI* which is specifically transcribed in rice roots. *MIKKI* contains multiple introns and has low coding potential, which is a strong sign of domestication to lncRNA. Intriguingly, the fourth intron of *MIKKI* is derived from an independent family of retrotransposon and the splicing of this intron generates a binding site for miR171 in the exon–exon junction. Despite the miR171-binding sequence, *MIKKI* mRNAs are not cleaved by miR171. The miR171-binding site of *MIKKI* does not perfectly base-pair with its cognate miRNA but has two mismatches at the positions where the cleavage is supposed to occur. It is very well-known that mismatches in the cleavage positions attenuate the cleavage activity of miRNA and is regarded as the signature of miRNA target mimic (Franco-Zorrilla et al., 2007; Yan et al., 2012; Reichel et al., 2015). Indeed, the knock-out mutants of *MIKKI* that had lost the target mimicking sequence showed higher levels of miR171, while overexpression of *MIKKI* resulted in the downregulation of miR171. miR171 targets the mRNAs encoding SCARECROW-Like (SCL) transcription factors which are critical regulators of root development (Wang et al., 2010). Therefore, *MIKKI* evolved from retrotransposons in rice and was positively selected to suppress miR171 in root. This in turn stabilizes the mRNAs of *SCLs* which are essential in the root development. Similarly, Quattro et al., 2017 also identified multiple TE-derived lncRNAs from *Brachypodium* genome that are able to interact with miRNAs, however, their target mimicry activities are yet to be confirmed.

## Concluding Remarks

Transposon-derived RNAs have been underestimated for a long time and their biological function have just started to be unveiled. The fact that TEs are repeated in the genome has significantly hampered the investigation of transposons so far. For example, the short length of the next-generation sequencing reads causes drastic ambiguity and imprecision in mapping the TE reads. Recent advance of the long read sequencing of PacBio (Disdero and Filée, 2017) and Oxford Nanopore (Debladis et al., 2017) is expected to overcome this shortcoming. In addition, due to the multiplicity of TEs in the genome and possible redundancy between them, the genetic analyses of TEs have been challenging. CRISPR-mediated mutagenesis has become more efficient and even a large deletion of an entire TE can be made by triggering the double-strand breaks in the flanking regions of the targeted TE (Gao et al., 2016; Ordon et al., 2017). Another option worth to consider is the population genetics approach. There is increasing number of available genome sequences and the number will grow exponentially as the sequencing cost drops. A large scale genome resequencing data analysis performed in the *Arabidopsis* natural accessions revealed that the TE landscape is very dynamic and the transcriptomic, epigenomic as well as phenotypic variations are attributed to TEs (Quadrana et al., 2016; Stuart et al., 2016). Taken all together, it seems that now is the best time to explore the hidden roles of TEs in plants by applying the new technologies developed recently.

## Author Contributions

The author confirms being the sole contributor of this work and approved it for publication.

## Conflict of Interest Statement

The author declares that the research was conducted in the absence of any commercial or financial relationships that could be construed as a potential conflict of interest.
